# The Geometry of the roots of the Brachial Plexus

**DOI:** 10.1111/joa.13270

**Published:** 2020-07-06

**Authors:** Ryckie G. Wade, Emily R. Bligh, Kieran Nar, Rebecca S. Stone, David J Roberts, Irvin Teh, Grainne Bourke

**Affiliations:** ^1^ Leeds Institute for Medical Research University of Leeds Leeds UK; ^2^ Department of Plastic and Reconstructive Surgery Leeds Teaching Hospitals Trust Leeds; ^3^ Division of Anatomy Leeds Institute of Medical Education University of Leeds Leeds UK; ^4^ Faculty of Medicine Dentistry & Health University of Sheffield Medical School Sheffield UK; ^5^ Faculty of Engineering University of Sheffield Sheffield UK; ^6^ School of Computing University of Leeds Leeds UK; ^7^ Leeds Institute of Cardiovascular and Metabolic Medicine University of Leeds Leeds UK

**Keywords:** anatomy, angle, Brachial plexus, diffusion tensor imaging, geometry, magnetic resonance imaging, microanatomy, pre‐ganglionic, root

## Abstract

Diffusion tensor magnetic resonance imaging (DTI) can be used to reconstruct the brachial plexus in 3D via tracts connecting contiguous diffusion tensors with similar primary eigenvector orientations. When creating DTI tractograms, the turning angle of connecting lines (step angle) must be prescribed by the user; however, the literature is lacking detailed geometry of brachial plexus to inform such decisions. Therefore, the spinal cord and brachial plexus of 10 embalmed adult cadavers were exposed bilaterally by posterior dissection. Photographs were taken under standardised conditions and spatially calibrated in MATLAB. The roots of the brachial plexus were traced from the dorsal root entry zone for 5 cm laterally using a 2.5‐mm^2^ Cartesian grid overlay. The trace was composed of points connected by lines, and the turning angle between line segments (the step angle) was resolved. Our data show that the geometry of the roots increased in tortuosity from C5 to T1, with no significant differences between sides. The 1^st^ thoracic root had the most tortuous course, turning through a maximum angle of 56° per 2.5 mm (99% CI 44° to 70°). Significantly higher step angles and greater variability were observed in the medial 2 cm of the roots of the brachial plexus, where the dorsal and ventral rootlets coalesce to form the spinal root. Throughout the brachial plexus, the majority of step angles (>50%) were smaller than 20° and <1% of step angles exceeded 70°. The geometry of the brachial plexus increases in tortuosity from C5 to T1. To reconstruct 99% of tracts representing the roots of the brachial plexus by DTI tractography, users can either customise the step angle per root based on our findings or select a universal threshold of 70°.

## Graphical abstract

To create 3D reconstructions of the brachial plexus by diffusion tensor magnetic resonance imaging, the geometry of the brachial plexus must be defined at high resolution and throughout its course. Our study traces the brachial plexus in ten cadavers to resolve the step/turning angles at a resolution of 2.5 mm^2^. We show that the tortuosity of the roots increases in the caudal direction and show that a step/turning angle of 70° is optimal.

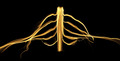

## 1 INTRODUCTION

**Figure 1:**
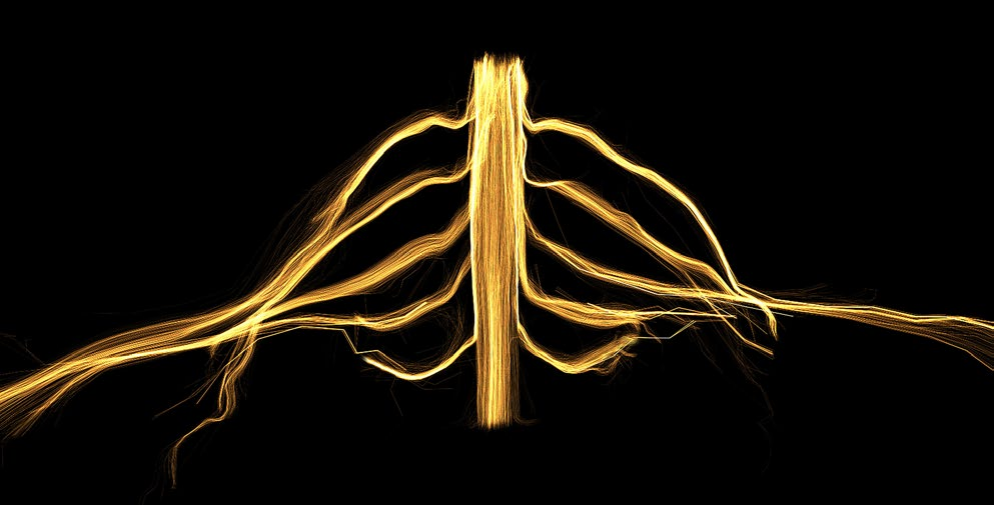
The spinal cord and brachial plexus of a healthy adult reconstructed from diffusion tensor magnetic resonance imaging, using example data acquired by our group

Magnetic resonance imaging (MRI) enables clinicians to visualise the postganglionic brachial plexus, but conventional sequences are unable to reconstruct images of rootlets and spinal roots (Wade *et a*l. 2019). Recent advances in the field of diffusion‐weighted MRI have overcome this problem through diffusion tensor imaging (DTI) which can reliably generate tracts of peripheral nerves in 3D (Schilling *et al*. 2019), including the brachial plexus (Figure 1 and Video S1).

**Figure 2:**
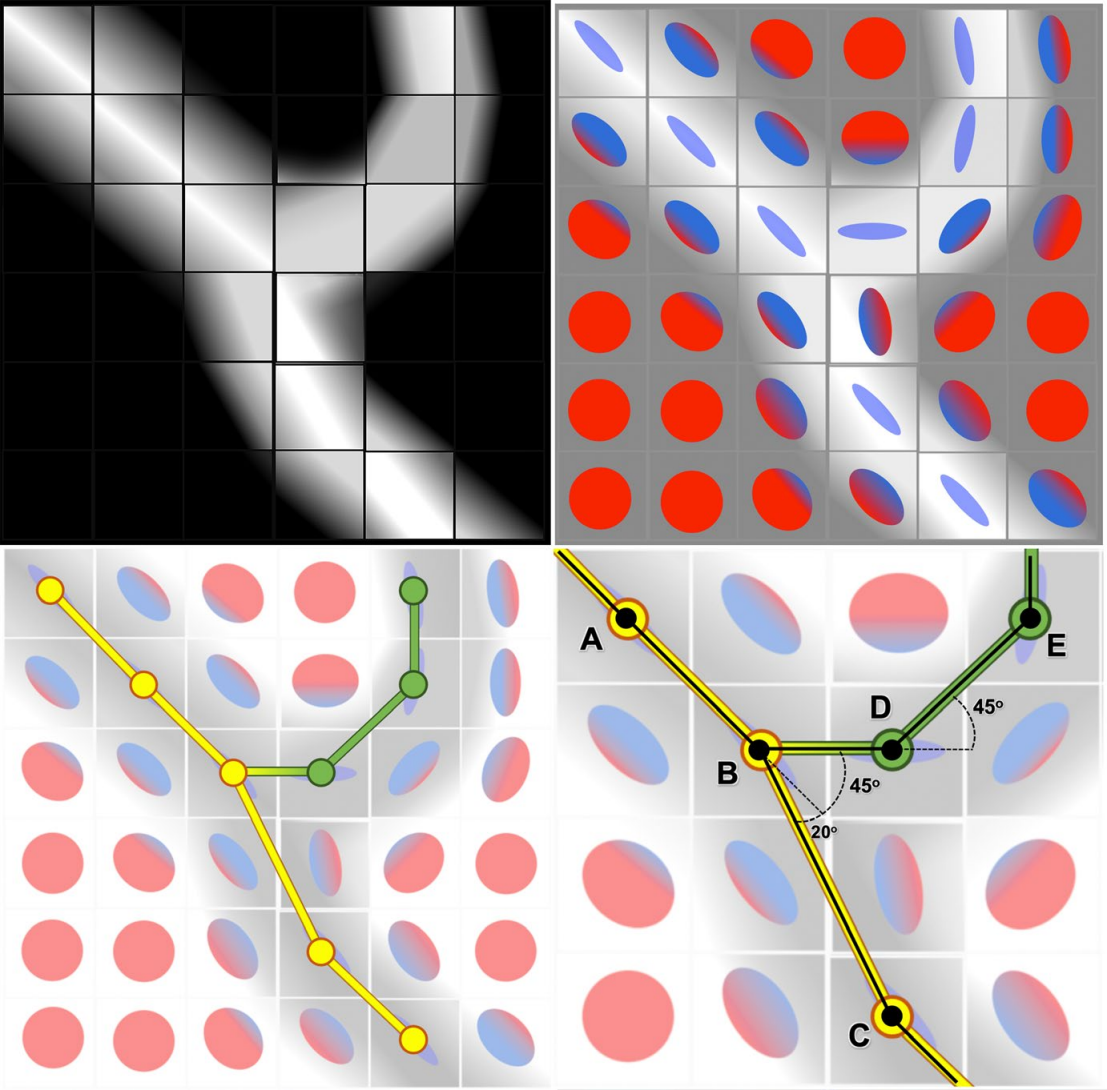
A simplification of diffusion tensor imaging (DTI) tractography. (a) A 2D artistic example of a diffusion‐weighted image through an imaginary bifurcating nerve (shown in black). In reality, the squares are cubes containing 3D information on the direction and magnitude of the diffusion of water. (b) Diffusion is modelled by a 3D vector (tensor) and simplified into a colour ellipsoid, whereby the colour denotes the direction and the shape describes the degree of directionality—a sphere represents isotropic diffusion whereas a thin ellipsoid represents anisotropic diffusion; healthy peripheral nerves have highly anisotropic diffusion. (c) Tracts (line segments) are propagated along voxels with locally aligned primary eigenvectors and a minimum fractional anisotropy. (d) A zoomed section of Panel (c) shows that users must specify the maximum angle allowed between new line segments (the step angle aka turning angle). To track the yellow portion of the nerve (line segment $\vec{ABC}$), a threshold of ≤25° would be sufficient but to track the green section of the nerve also (line segment $\vec{ABDE}$), a higher step angle would be needed

A simplification of the process involved in creating a tractogram from DTI data is shown in Figure 2 and demonstrates the importance of the turning/step angle in generating valid tracts (Parizel *et al*. 2007). The step angle is an important factor to consider when tracking tortuous structures across adjacent voxels (rather than microscopic structures which may be tortuous at the intra‐voxel level). If the user selects a step angle which is excessively high, then numerous non‐valid and looping tracts may be produced. If the step angle is too low for a structure which is tortuous across adjacent voxels, then tracts will not propagate (Figure 2d). Therefore, when performing tractography on tortuous structures (such as the brachial plexus), it is important to select a step angle which is sufficient to propagate valid tracts but not higher than necessary, to minimise non‐valid tracts.

Two anatomical studies have investigated the geometry of the brachial plexus in adult cadavers (Xiang *et al*. 2008; Zhong *et al*. 2017). Xiang *et al*. (2008) summarised the microanatomy of the dorsal root entry zone and dorsal rootlets, showing that the average angle between the inferior rootlets of T1 and the median sulcus of the spinal cord was 66^o^ in the coronal plane; this angle decreased in a cranial direction with the C4 root branching at 20°. Xiang *et al*. (2008) provided a valuable insight into the step angle needed to propagate tracts of the rootlets, but their study only considered the most proximal 1.5 cm (the rootlets) of the brachial plexus. More recently, Zhong *et al*. (2017) performed similar dissections although they only acquired a single measurement of the angle between the median sulcus of the spinal cord and each spinal root at the level of the dorsal root ganglion in the coronal plane, which substantially underestimates the complexity of the geometry of the roots.

Anatomical studies provide limited information on the geometry of the brachial plexus, which manifest in the wide variety of step angles (14° to 70°) used in DTI studies of the brachial plexus to date (Vargas *et al*. 2010; Tagliafico *et al*. 2011; Gasparotti *et al*. 2013; Oudeman *et al*., 2018; Wade *et al*., 2020; Su *et al*. 2019). The need for precise and detailed geometric information, which can be translated to clinical diffusion tensor tractography, represents the rationale for this study.

## 2 METHODS

This anatomical study, which was conducted between June and August 2019, included ten adult cadavers (of mean age 85 years) donated to the Leeds Medical School. Each donor had given written consent to donation and to the use of their body for research purposes. Approval for the work was granted by the University of Leeds Anatomy Access Committee (Reference 200619). The study complied with the Human Tissue Act (2004).

### 2.1 Objectives

The primary objective was to detail the geometry of the rootlets and spinal roots of the brachial plexus in two dimensions (in the coronal plane).

### 2.2 Embalming process

Donors were perfused through the left common carotid or femoral artery with approximately 30–50 L of a premixed preservation fluid consisting of 1.6% formaldehyde, 3.8% methanol, 9% water, 10% phenol and 75.6% ethanol. Cadavers were stored at 4°C until dissection.

### 2.3 Dissection

**Figure 3:**
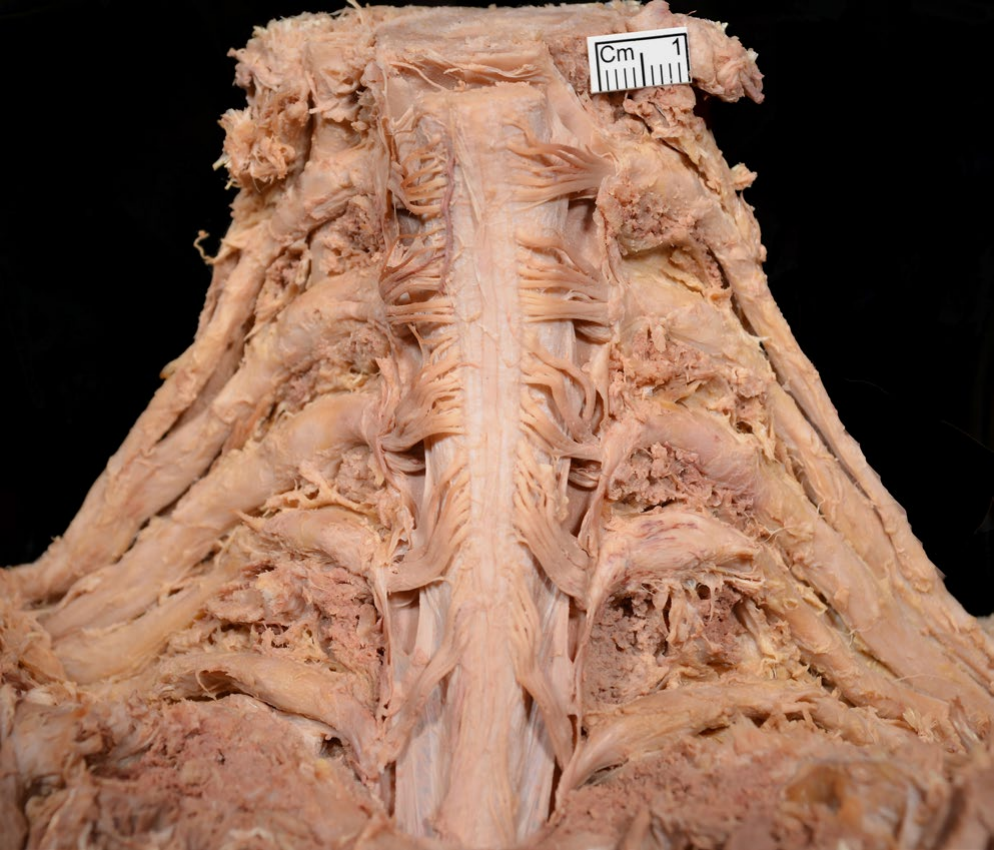
A posterior dissection of the spinal cord and roots of the brachial plexus

The spinal cord and brachial plexus were demonstrated bilaterally via a posterior approach, with osteotomies through the pedicles of C4‐T1. The dorsal and ventral rootlets have an identical length, and angles in both the coronal and axial planes within the spinal canal (Zhong *et al*. 2017), so to preserve the exact course of dorsal rootlets and spinal roots, the anterior (ventral) rootlets were not dissected (Figure 3). None of the cadavers had any relevant pathology.

### 2.4 Photography and image analysis

All specimens were photographed under fixed conditions using the same camera consistently positioned orthogonal to the imaging plane at 30 cm from the C7 cord level to the lens. A ruler with 1 mm increments was included in the field of view adjacent to the roots. Images were imported to MATLAB R2019b (The MathWorks, Inc.), calibrated and a 2.5‐mm^2^ cartesian grid overlaid. Twenty‐one points were placed at 2.5‐mm intervals along the left‐right direction, starting from the respective dorsal root entry zone and extending for 5 cm along each of the C5‐T1 nerve roots (Figure S1). We chose to trace the mid‐point of the rootlets/roots because this approach provided the lowest possible estimate of the step angle through the course of the nerves. Further, when tractography algorithms are tracking into nearby voxels of similar anisotropy, tracts will preferentially propagate into the voxel with the lowest step angle. The line segments between points were plotted, and the step angles (Figure 2d) and gradient of each line were resolved.

### 2.5 Statistical analysis

Data were analysed in Stata/MP v15 (StataCorp LLC). As this is research was concerned with generating estimates of the normal geometry of the roots of the brachial plexus and there were no formal hypotheses to test, there was no role for a power calculation. The sample size was based upon the availability of cadavers and staff time within the dissection room. Step angles are skewed so summarised by the geometric mean and 99% confidence intervals (CI). Non‐parametric regression was used to model step angle data. To estimate how the geometry differed between the five roots (C5 to T1), between sides (left and right) and between individuals (ten cadavers), these were modelled as categorical fixed‐effects in multivariable non‐parametric regression. To quantify the tortuosity of roots, the residual variance from the multivariable non‐parametric regression was calculated and summarised per root.

## 3 RESULTS

**Figure 4:**
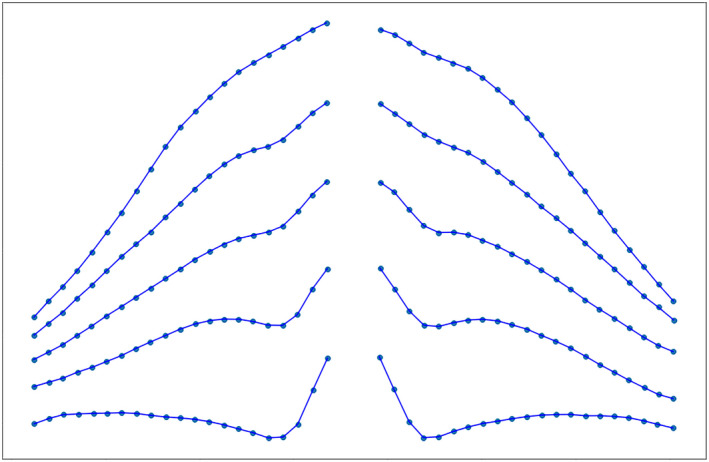
A summary plot of the course of the roots of the brachial plexus in 10 cadavers

**Figure 5:**
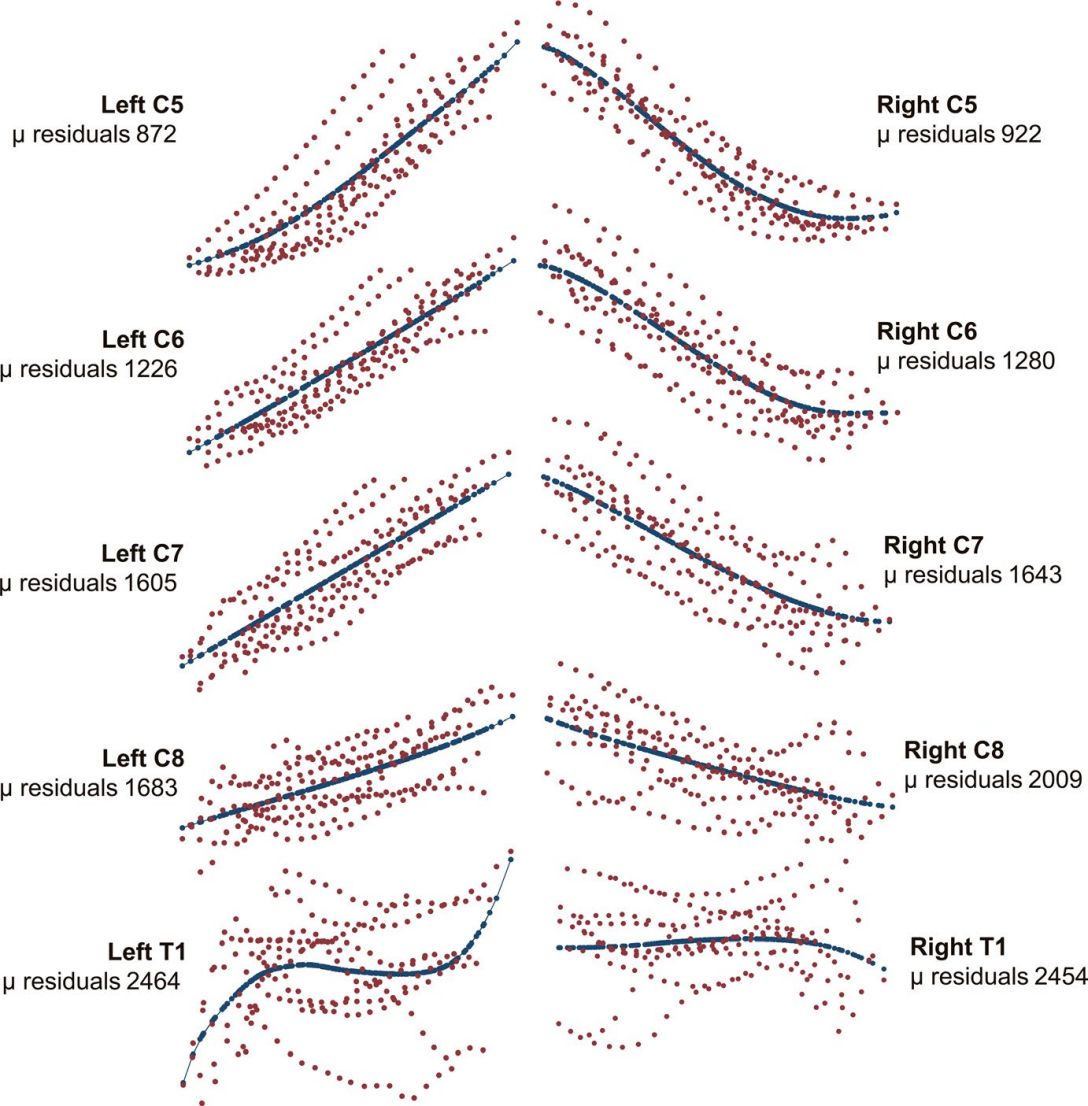
Scatter plots of traces for each root (red dots) with a non‐parametric regression line of fit (blue). The mean (μ) of the residual variance is provided to quantify the tortuosity, whereby a higher mean residual variance implies a more tortuous nerve

The summary trace of ten cadavers (Figure 4) and non‐parametric regression plots in Figure 5 demonstrate that the tortuosity of spinal roots increases in the caudal direction (Table 1; *p* < 0.001). There was no statistically significant difference in the geometry of left versus right roots (*p* = 0.170).

**Table 1:** The step angles of the roots of the brachial plexus averaged across samples

Root	Mean step angle in degrees (99% CI)	Maximum step angle in degrees (99% CI)
C5	7 (6, 9)	20 (15, 25)
C6	8 (7, 9)	25 (21, 30)
C7	9 (7, 12)	32 (23, 43)
C8	11 (10, 13)	44 (34, 57)
T1	12 (11, 13)	56 (44, 70)

**Figure 6:**
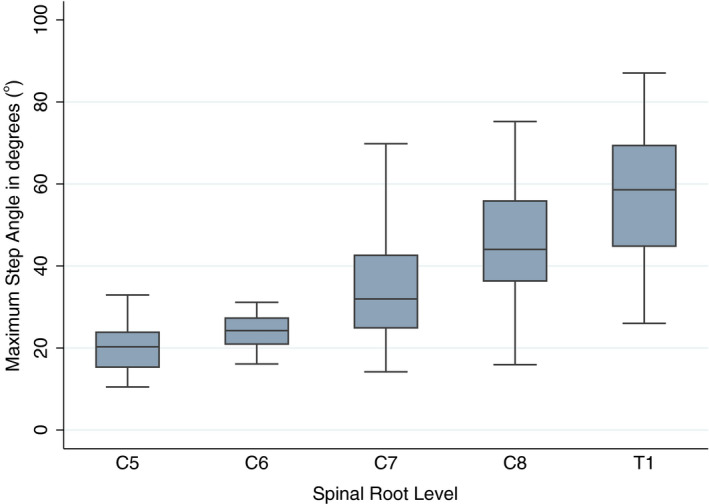
Boxplots showing the maximum angles (averaged across cadavers) for each root of the brachial plexus

The C5 and C6 roots had very similar geometry (*p* = 0.906), with little variability throughout their course and a small step angle (Table 1 and Figure 5). Conversely, the T1 root had the greatest maximum step angle with a mean of 56° (99% CI: 44° to 70°) as well as the greatest variability throughout its course (Figures 5 and 6). Compared to the C5/6 roots, for every 2.5 mm the C7 root turned an additional 2° (99% CI: 1° to 4°); the C8 turned an additional 4° (99% CI: 2° to 5°); and the T1 root turned an additional 4° (99% CI: 3° to 5°).

**Figure 7:**
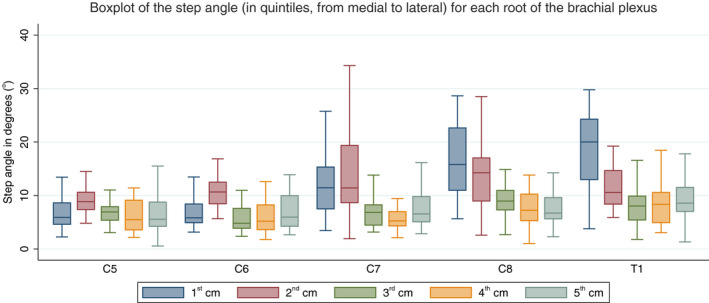
Boxplots of the angles of each root, divided into quintiles (from medial to lateral) showing that the majority of the variability in the measured angles is observed within the medial 2 cm

The majority of the variability in the course of the C7, C8 and T1 roots was observed in the medial two quintiles (medial 2 cm) which turned an addition 5° per 2.5 mm [99% CI: 4° to 6°] compared to the lateral three quintiles (Figure 7). There was no statistically significant difference between the step angles measured in the 1st and 2nd quintiles (*p* = 0.926). There were no statistically significant differences between the step angles measured in the 3rd, 4th or 5th quintiles. This implies that the majority of the tortuosity is observed within the intradural and intraforaminal portions of the brachial plexus.

As some sections of nerves are more vertically oriented than others (e.g. the rootlets of the T1 are more vertical than the C5 rootlets), we correlated step angles with the gradients of the line segments to understand whether we had introduced a bias in our methods, given that the effective resolution was modified. No statistically significant association was observed (*r* = 0.017, *p* = 0.065; Figure S3) implying that no bias was introduced by our methods.

Translating these measurements to clinical DTI tractography, Figure S2 shows that 50% of step angles are ≤20° and fewer than 1% exceed 70°. Therefore, to plot 99% of tracts representing the roots of the brachial plexus, a step angle of 70° is likely to be sufficient to render tracts of 99% of roots. To demonstrate this concept, the summary plot from all cadavers is overlaid onto an example tractogram derived from a healthy adult (Figure S4).

## 4 DISCUSSION

When reconstructing the brachial plexus using diffusion tensor imaging tractography, a step angle of 70° is likely to enable the propagation of 99% of tracts representing the roots. Our findings agree with the two cadaveric studies of the geometry of the brachial plexus whereby *Xiang et al*. (2008) showed that the rootlets of the T1 had a step angle of 66° over 15 mm and Zhong *et al*. (2017) showed that both the dorsal and ventral rootlets had a step angle of approximately 70°. However, our study adds more detailed information to the literature regarding the microscopic geometry of the pre‐ and postganglionic brachial plexus which can be readily translated to clinical imaging. Our findings might be applied in one of two ways: (a) when tractography software requires a fixed step angle for a session of tractography, then the upper 99% confidence interval value (70°) could be used, accepting that some false tracts may be generated, necessitating topology informed pruning (Yeh *et al*. 2019), or (b) if bespoke step angles can be customised on the fly, then the upper value of the root‐specific 99% confidence intervals could be selected (e.g. 25° for C5, 30° for C6, 43° for C7, 57° for C8 and 70° for T1; Table 1); this approach would minimise the probability of generating false tracts whilst maximising the probability of representing the true geometry of the roots.

A DTI tractogram of the brachial plexus (Figure 1) is the result of a multistep process, with numerous assumptions and uncertainties (Jones, Knösche and Turner, 2013). The principal eigenvector of the tensor cannot be assumed to be an accurate representation of the actual fibre orientation(s) because it cannot resolve crossing, diverging/converging, twisting or kinked fibres so is likely to generate errors somewhere along the fibre path. Further, the relationship between DTI metrics and the microstructure of peripheral nerves, in the context of both health and disease, is not well understood and is the topic of ongoing research. Finally, as with many complex processes, there are numerous factors which can alter the quality of the diffusion data and metrics, including the following: the disease trajectory and scanning time, patient positioning, coil selection, field strength and inhomogeneities, pulse sequence parameters, data pre‐processing, mathematical models for tensorial calculations, regions of interest and the algorithms for tractography and derivation of DTI parameters. A small alteration in any of these parameters can affect the output and so it is important that choices in the clinical imaging pipeline are grounded in evidence.

Diffusion tensor magnetic resonance imaging tractography has gained attention globally given its unparalleled ability to generate high fidelity maps of neural pathways from non‐invasive imaging and provide objective proxy measures of nerve health. However, to be able to differentiate healthy from diseased or injured sections of nerve, it is necessary to define the normative DTI parameters and tractography conditions for the brachial plexus. To date, six studies report the findings of DTI tractography of the brachial plexus in healthy adults (Vargas *et al*. 2010; Tagliafico *et al*. 2011; Gasparotti *et al*. 2013; Oudeman *et al*., 2018; Wade *et al*., 2020; Su *et al*. 2019; Table S1) but a wide array of step angles was used to reconstruct tracts representing the pre‐ and postganglionic brachial plexus. Vargas *et al*. (2010) used a 30° step angle and reconstructed the C5‐T1 roots in all volunteers, although the C5 roots were not apparent in their published tractograms. Gasparotti *et al*. (2013) used a step angle of 35–45^o^ to propagate tracts of the C5‐T1 roots in all individuals; however, their published tractograms showed the T1 tracts terminating close to the spinal cord. Wade *et al*. (2020) used a step angle of 35° although 4% of C5–C8 and 46% of T1 roots did not propagate. Oudeman *et al*. (2018) used a step angle of 14° which reconstructed all C5‐C8 tracts but failed to propagate T1 tracts in 52%. Neither Tagliafico *et al*. (2011) or Su *et al*. (2019) described the step angle used for tractography or the proportion of tracts generated; their articles contained data on the C5‐C8 roots and did not describe why the T1 root data were excluded. We hope that future DTI tractography studies can produce more reliable tractograms using the information within our report.

### 4.1 Limitations

A 2D study of the geometry of the brachial plexus incompletely describes its complex course and 3D modelling would (in theory) be superior; however, (a) the techniques for generating 3D structure from motion are immature and would require the attachment of circumferential beacons, meaning further dissection and perturbed anatomy, (b) the angles through which the roots of the plexus turn in the axial and sagittal planes are substantially less than in the coronal plane. Therefore, thresholding would need to be based on the coronal angles, rendering measurements of angles in any other 2D plane irrelevant. In Figure S4, we overlay the summary plot from all cadavers onto a DTI tractogram from a single healthy adult, so formal assessments of agreement cannot be made. Ideally, DTI would be acquired on a recently deceased non‐embalmed donor (Haakma *et al*. 2016) and subsequent dissection would facilitate an assessment of agreement between the tractogram and anatomy. The translation of our findings may be limited because the geometry of the plexus in younger individuals may be different to the adults we studied, and furthermore, the normal variation within the population may not be adequately captured by our relatively small sample.

## 5 CONCLUSIONS

The geometry of the roots of the brachial plexus increases in complexity from C5 to T1. When reconstructing the roots of the brachial plexus using diffusion tensor imaging tractography, a step angle of 70° is likely to plot 99% of tracts representing the roots, based on the limited sample size of this study.

## CONFLICT OF INTERESTS

None.

## ETHICAL APPROVAL

Approval for this work was granted by the University of Leeds Anatomy Access Committee.

## Supporting information

Figure S1

Figure S2

Figure S3

Figure S4

Table S1

Video S1

## Data Availability

The data that support the findings of this study are available from the corresponding author upon request.
