# Supplementary material for: The Geometry of the roots of the Brachial Plexus
Source: J Anat. 2020 Jul 6;237(6):999–1005. doi: 10.1111/joa.13270 (PMC7704236; doi:10.1111/joa.13270)
Supplement: Supplementary file 5 — Table S1 [file JOA-237-999-s005.docx]

**Supplementary Table 1.** Characteristics of the studies using DTI to visualise healthy roots of the brachial plexus

| **Study** | **N** | **Field Strength (Tesla)** | **Sequence** | **Pre-processing** | **ROI position** | **ROI size** | **Tractography conditions** | **Tracts propagated** | **Datasets excluded** |
| --- | --- | --- | --- | --- | --- | --- | --- | --- | --- |
| (Gasparotti *et al. (*2013) | 28 | 1.5 | ssEPI: b0/900 s/mm^2^), 12 directions, 5 averages, TE 90ms, TR 9200ms, 2.2mm isotropic voxels. | FSL: Motion & eddy correction using FMRIBs Diffusion Toolbox before concatenation | 10 ROIs, one at each neural foramina | 5mm diameter | 2^nd^ order RK, 35-45^o^ step angle | All C5-T1 | Not described |
| Vargas *et al. (*2010) | 6 | 1.5 | ssEPI: b0/900 s/mm^2^), 30 directions, no repetitions, TE 78ms, TR 9000ms, 2mm isotropic voxels | Not described | “Several places on the spinal cord, trunks and cords” | 2mm^2^ | Siemens Neuro 3D: 4^th^ order RK, variable FA threshold (0.15 +/- 0.05) 30^o^ step angle, 0.9mm step length, | All C5-T1 | Not described |
| Tagliafico *et al. (*2011) | 40 | 3T | ssEPI: b0/1000s/mm^2^, 32 directions, averaging not described, TE “minimum”, TR 16675ms, 2mm slice thickness. | Not described | Not described | 2mm^2^ | GE Functool v6.3.1: Variable FA threshold (0.15 +/- 0.05), 1mm step length | Not described | Not described |
| Oudeman *et al.* (2018) | 30 | 3T | ssEPI: b0/800s/mm^2^, 15 directions, TE 77ms, TR 5969ms, 6 averages, 3mm isotropic. | ﻿DTItools for Mathematica v11.3: ﻿Rician noise suppression, eddy and motion correction using the b0s  Tensor calculation = ﻿weighted linear least-squares | Ten ROIs (1 per root) close to the ganglia | Not described | VIST/e; An unspecified “deterministic tracking algorithm”, FA threshold 0.1-0.8, 14^o^ step angle, 0.15mm step length, 1mm^2^ seed density, 3cm minimum tract length | All C5-C8 roots 48% of T1 tracts | Not described |
| Su *et al.* (2019) | 163 | 3T | rsEPI: 0/900 s/mm^2^, 20 directions, 4 averages, TE 92ms, TR 6000ms, 2x2x3mm voxels | Not described | Postganglionic roots 1cm lateral to the DRG | Not described | Not described | Not described | 5 (3%) due to motion |
| Wade *et al. (*2020) | 17 | 3 | ssEPI: b0,1000 s/mm^2^, 20 directions, 4 averages, TE 66ms, TR 4300ms, 2.5mm isotropic. | No corrections  Tensor calculation = ﻿weighted linear least-squares | Lateral recess of the intervertebral foramina | 10mm^2^ | Siemens Neuro 3D: 4^th^ order RK, FA threshold 0.06, 35^o^ step angle, 1.1mm step length | 96% of C5-C8  50% of T1 | None |

DRG = dorsal root ganglia, reEPI = readout segment echo planar imaging; ssEPI = single shot echo planar imaging; RK = Runge-Kutta; T = Tesla TE = echo time; TR = repetition time
